# Chemical Composition and Insecticidal Activity of Essential Oils from *Zanthoxylum dissitum* Leaves and Roots against Three Species of Storage Pests

**DOI:** 10.3390/molecules20057990

**Published:** 2015-05-04

**Authors:** Cheng-Fang Wang, Kai Yang, Chun-Xue You, Wen-Juan Zhang, Shan-Shan Guo, Zhu-Feng Geng, Shu-Shan Du, Yong-Yan Wang

**Affiliations:** 1 Beijing Key Laboratory of Traditional Chinese Medicine Protection and Utilization, Beijing Normal University, No.19, Xinjiekouwai Street, Beijing 100875, China; E-Mails: wangchengfang@mail.bnu.edu.cn (C.-F.W.); yangk_1988@mail.bnu.edu.cn (K.Y.); youchunxue@mail.bnu.edu.cn (C.-X.Y.); zwj0729@mail.bnu.edu.cn (W.-J.Z.); ssdyx1990@163.com (S.-S.G.); gengzhufeng@bnu.edu.cn (Z.-F.G.); narcissus09@126.com (Y.-Y.W.); 2 China CDC Key Laboratory of Radiological Protection and Nuclear Emergency, National Institute for Radiological Protection, Chinese Center for Disease Control and Prevention, Beijing 100088, China

**Keywords:** *Zanthoxylum dissitum*, essential oil, contact activity, stored product pests

## Abstract

This work aimed to investigate chemical composition of essential oils obtained from *Zanthoxylum dissitum* leaves and roots and their insecticidal activities against several stored product pests, namely the cigarette beetle (*Lasioderma serricorne*), red flour beetle (*Tribolium castaneum*) and black carpet beetle (*Attagenus piceus*). The analysis by GC-MS of the essential oils allowed the identification of 28 and 22 components, respectively. It was found that sesquiterpenoids comprised a fairly high portion of the two essential oils, with percentages of 74.0% and 80.9% in the leaves and roots, respectively. The main constituents identified in the essential oil of *Z*. *dissitum* leaves were *δ*-cadinol (12.8%), caryophyllene (12.7%), *β*-cubebene (7.9%), 4-terpineol (7.5%) and germacrene D-4-ol (5.7%), while humulene epoxide II (29.4%), caryophyllene oxide (24.0%), diepicedrene-1-oxide (10.7%) and *Z,Z,Z*-1,5,9,9-tetramethyl-1,4,7-cycloundecatriene (8.7%) were the major components in the essential oil of *Z*. *dissitum* roots. The insecticidal activity results indicated that the essential oil of *Z*. *dissitum* roots exhibited moderate contact toxicity against three species of storage pests, *L. serricorne*, *T. castaneum* and *A. piceus*, with LD_50_ values of 13.8, 43.7 and 96.8 µg/adult, respectively.

## 1. Introduction

Traditional Chinese Medicines (TCMs) are gaining popularity as a form of complementary and alternative medicine [1]. Over the past decades, research on TCMs has mainly focused on their efficacy [2,3,4,5]. However, studies concerning on the storage and stability of TCMs are insufficient. TCMs mainly include Chinese medicinal herbs and animals parts, which are very vulnerable to insect attack during storage. The antagonistic storage method is one of traditional TCM conservation methods and has been in use for a long time, playing an important role in the protection and utilization of TCM resources. It mainly utilizes some Chinese Medicinal Materials (CMMs) with special volatile odors to prevent another CMM from suffering insect attacks. With the increasing attention of ecological protection and medication security, it is believed that this sustainable control method for stored-product pests would have broad application prospects in the future. In order to develop this traditional method of prevention and control of storage pests, we have established a screening program for new agrochemicals from CMMs [6,7,8,9,10], in which we focus on the volatile substances due to their major role in the antagonistic storage process. During this screening process, the essential oil of *Zanthoxylum dissitum* Hemsl was found to possess insecticidal activity against three species of stored-product pests, the cigarette beetle (*Lasioderma serricorne* Frbricius), red flour beetle (*Tribolium castaneum* Herbst) and black carpet beetle (*Attagenus piceus* Oilver), which are dominant populations in the stored CMM insect community and also widely distributed pests that destroy stored products worldwide [11,12,13].

*Z. dissitum*, a climbing woody vine, belongs to the family Rutaceae and is mainly distributed in Guangxi, south of Shanxi and Gansu, Guizhou, and west Sichuan Province [14]. The roots, stems and leaves are used as traditional Chinese medicinal herbs for the treatment of many painful conditions such as sprains, lower back and leg pain, and fractures [15]. Phytochemical studies on *Z. dissitum* have revealed the presence of coumarins, alkaloids, amides and phytosterols [16,17,18], but to the best of our knowledge there are no reports available on the essential oil of *Z. dissitum* or its insecticidal activity against stored product insects. Thus, the chemical composition of essential oils derived from *Z. dissitum* leaves and roots and their insecticidal activities towards three species of stored-product pests are presented in this paper for the first time. It was expected that this work would provide more theoretical background for the conception of useful antagonistic storage methods and comprehensive utilization of TCM resources.

## 2. Results

### 2.1. Chemical Composition of the Essential Oils

The GC-FID and GC-MS analysis results for the *Z*. *dissitum* essential oils are summarized in Table A1. The *Z*. *dissitum* leave and root essential oil yields were 0.9% and 0.3% (*v*/*w*), respectively. The density of the concentrated essential oils was determined as 0.8 and 0.9 g mL^−1^, respectively. A total of 28 components were identified in the essential oil of *Z*. *dissitum* leaves, accounting for 92.1% of the total oil and the main constituents were *δ*-cadinol (12.8%), caryophyllene (12.7%), *β*-cubebene (7.9%), 4-terpineol (7.5%) and germacrene D-4-ol (5.7%), followed by *τ*-muurolol (4.5%), and ubenol (4.3%) (Table A1), while the GC-MS data revealed a total of 22 identified constituents which could represent 94.0% of the total essential oil from *Z*. *dissitum* roots, in which the major components were humulene epoxide II (29.4%), caryophyllene oxide (24.0%), diepicedrene-1-oxide (10.7%) and *Z,Z,Z*-1,5,9,9-tetramethyl-1,4,7-cycloundecatriene (8.7%), followed by cubenol (4.1%), estragole (3.9%) and linalool (3.8%) (Table A1).

### 2.2. Insecticidal Activities

Percent fumigant/contact mortality of each beetle species after 24 h exposure to increasing concentrations of the two essential oils is shown in Table 1. 

**Table 1:** The corrected mortality of essential oils of *Z*. *dissitum* leaves and roots against three species of storage pests in fumigant/contact toxicity assays.

Treatment	Concentration (%)	*L. serricorne* Adults		*T. castaneum* Adults		*A*. *piceus* Larvae		
		Fumigant Toxicity	Contact Toxicity	Fumigant Toxicity	Contact Toxicity		Fumigant Toxicity	Contact Toxicity
Control	0	0	0	0	0		0	0
*Z*. *dissitum* leaves	50	40.0 ± 10.0	33.3 ± 15.3	13.3 ± 11.6	53.3 ± 5.8		13.3 ± 5.8	22.0 ± 8.4
	10	20.0 ± 10.0	26.7 ± 11.6	3.3 ± 5.8	36.7 ± 5.8		0	10.0 ± 10.0
	2	10.0 ± 10.0	20.0 ± 10.0	0	23.3 ± 15.3		0	0
*Z*. *dissitum* roots	50	10.0± 10.0	100 ± 0.0	13.3 ± 15.3	100 ± 0.0		3.3 ± 6.4	100 ± 0.0
	10	3.3 ± 5.8	88.9 ± 11.1	13.3 ± 5.8	53.3 ± 5.8		0	36.0 ± 11.4
	2	0	14.8 ± 6.4	0	10.0 ± 10.0		0	0

**Figure 1:**
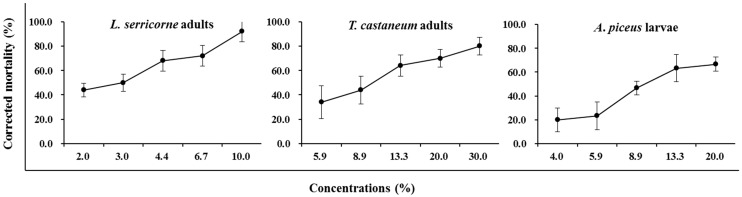
Contact toxicity of the essential oil of *Z*. *dissitum* roots against three species of storage pests.

The essential oils of *Z*. *dissitum* leaves and roots exhibited weak fumigant toxicity against *L. serricorne* adults, *T. castaneum* adults and *A*. *piceus* larvae, with the highest concentration tested (50%) causing less than 50% mortality. In the contact toxicity assays, the highest concentration (50%) of essential oil from roots caused 100% beetle mortality, while the percent mortality caused by 50% concentration of essential oil from leaves was only 33% in *L. serricorne* adults, 53% in *T. castaneum* adults and 22% in *A*. *piceus* larvae, respectively. The contact toxicity of essential oil from the roots was thus stronger than that of the leaves. In this context, we determined the appropriate testing concentrations of the essential oil of *Z*. *dissitum* roots to the three species of insects and calculated the LD_50_ values by using Probit analysis, with the results listed in Table 2. The three species of storage pests treated by the essential oil of *Z*. *dissitum* roots revealed different sensitivity, with LD_50_ values of 13.8, 43.7 and 96.8 µg/adult, respectively. In Figure 1, it can be seen that the contact toxicity of essential oil from roots towards *L. serricorne* adults, *T. castaneum* adults and *A*. *piceus* larvae was concentration-dependent.

**Table 2:** Contact toxicity of the essential oil of *Z*. *dissitum* roots against three species of storage pests.

Insect	Treatment	Concentration (%)	LD_50_ (µg/adult)	95% FL *	Chi Square (χ^2^)
*L. serricorne* adults	*Z.dissitum*	2.0–10.0	13.8	9.6–17.5	9.4
	Pyrethrins ^a^	-	0.2	-	-
*T. castaneum* adults	*Z.dissitum*	5.9–30.0	43.7	32.6–54.2	9.7
	Pyrethrins ^a^	-	0.3	-	-
*A*. *piceus* larvae	*Z.dissitum*	4.0–20.0	96.8	73.7–134.2	10.1
	Pyrethrins	0.2–1.0	5.9	4.6–8.6	35.7

* Fiducial limits; ^a^ data from Wang *et al*. [8].

## 3. Discussion

### 3.1. Chemical Composition of the Essential Oils

As shown in Table A1, the essential oils from two parts of the plant consisted mainly of sesquiterpenoids, which accounted for 74.0% in the leaves and 80.9% in the roots. However, among the chemical components of the two essential oils, there were only nine identical constituents, and the percentage of most of the same components showed great differences. These observed differences in chemical composition and content between the essential oils of leaves and roots could be due to the different effects of environmental factors (such as sunlight, water and soil) on the aerial parts and underground parts, or may result from different metabolic pathways in the plant. These variations of chemical composition may also lead to different biological activities.

### 3.2. Insecticidal Activities

During the fumigant toxicity tests, the essential oils from *Z*. *dissitum* leaves and roots showed weak toxicity to *L. serricorne* adults, *T. castaneum* adults and *A*. *piceus* larvae (Table 1), even though some components of the two essential oils such as caryophyllene oxide, 4-terpineol, estragole and linalool were found to possess fumigant toxicity against *T. castaneum* adults in previous reports [19,20,21]. This might be due to the high content of sesquiterpenoids and their relative distribution ratio in the essential oils from *Z*. *dissitum* leaves and roots. This suggested that the majority of the sesquiterpenoids in the *Z*. *dissitum* essential oils demonstrated antagonistic effects in the fumigant toxicity process.

**Figure 2:**
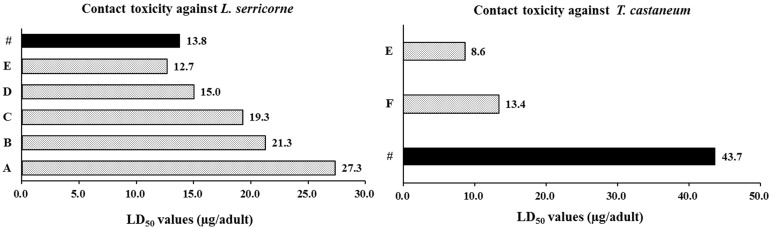
Comparison of the LD_50_ values with literatures. #, *Z. dissitum* roots; A, *Litsea cubeba*; B, *Cinnamomum camphora*; C, *Myristica fragrans*; D, *Alpinia blepharocalyx*; E, *Artemisia stolonifera*; F, *Liriope muscari*.

The essential oils of *Z*. *dissitum* roots showed strong contact toxicity against *L. serricorne* adults, *T. castaneum* adults and *A*. *piceus* larvae (Table 1 and Table 2). When compared with the positive control pyrethrins, the essential oil demonstrated 57, 168 and 16 times less acute toxicity against the three species of storage pests (Table 2). However, compared with the other essential oils reported in previous studies (Figure 2), the essential oil of *Z*. *dissitum* roots exhibited stronger contact toxicity against *L. serricorne*, e.g., essential oils of *Litsea cubeba* (LD_50_ = 27.3 μg/adult) [22], *Cinnamomum camphora* (LD_50_ = 21.3 μg/adult) [23], *Myristica fragrans* (LD_50_ = 19.3 μg/adult) [7]. The essential oil showed similar contact toxicity to the essential oils of *Alpinia blepharocalyx* (LD_50_ = 15.0 μg/adult) and *Artemisia stolonifera* (LD_50_ = 12.7 μg/adult) [24,25]. Meanwhile, the essential oil of *Z*. *dissitum* roots was found to have weaker toxicity to *T. castaneum* in comparison with the essential oils of *Artemisia stolonifera* (LD_50_ = 8.6 μg/adult) and *Liriope muscari* (LD_50_ = 13.4 μg/adult) [25,26].

## 4. Experimental Section

### 4.1. Plant Material and Extractions

Fresh leaves (4.0 kg) and roots (5.0 kg) of *Z*. *dissitum* were collected at November 2011 from Yulin (22.67°N latitude and 110.14°E longitude), Guangxi Province, China. The plant was identified by Prof. Yin, H.B. (College of Pharmacy, Liaoning University of Traditional Chinese Medicine, Dalian, China) and a voucher specimen (BNU-CMH-Dushuahan-2011-11-19-023) was deposited at the College of Resources Science & Technology, Beijing Normal University. The leaves and roots were air-dried for one week and ground to a powder. Then the powder was subjected to hydrodistillation for 6 h using a Clevenger-type apparatus. Anhydrous sodium sulfate was used to remove water after extraction. The essential oils were stored at 4 °C for subsequent experiments.

### 4.2. Gas Chromatography and Mass Spectrometry

Gas chromatographic (GC) analysis was performed on an Agilent 6890N instrument equipped with a flame ionization detector and an HP-5MS (30 m × 0.25 mm × 0.25 μm) capillary column. The column temperature was programmed at 50 °C for 3 min then increased at 10 °C/min until the final temperature of 290 °C was reached, where it was held for 20 min. The injector temperature was maintained at 250 °C. The samples (1 μL, dilute to 1% with *n*-hexane) were injected, with a split ratio of 1:50. Helium was used as carrier gas at a flow rate of 1.0 mL min^−1^. Percentage composition of individual components was calculated on the basis of peak area without using correction factors. The chemical constituents of essential oil were identified on an Agilent Technologies 5973N mass spectrometer. Various components were identified by comparison of their retention indices with literature values [27,28], computer matching against the library spectra (NIST 05, Wiley 275 and Adams Library). The retention indices were determined in relation to a homologous series of *n*-alkanes (C_8_–C_24_) under the same operating conditions. 

### 4.3. Insects

*L. serricorne* and *T. castaneum* were obtained from laboratory cultures maintained in the dark in incubators at 29–30 °C and 70%–80% relative humidity (RH). Both of them were reared on wheat flour mixed with yeast (10:1, *w*/*w*) at 12%–13% moisture content. The unsexed adults used in all the experiments were about 1–2 weeks old, and the average weight of per adult insect was 1.9 mg (*L. serricorne*) and 1.7 mg (*T. castaneum*), respectively. The larvae of *A*. *piceus* were obtained from the warehouse of Traditional Chinese Medicinal Materials in the laboratory and were about 2–3 months old, had an average weight of 6–7 mg. Its cultures condition was the same as *L. serricorne* and *T. castaneum*. All containers housing insects used in experiments were made escape proof with a coating of polytetrafluoroethylene (Sino-rich^®^, Beijing Sino-rich Tech Co., Ltd., Xuanwu District, Beijing, China).

### 4.4. Fumigant Toxicity

The fumigant toxicity of the essential oils against the three species of insects was measured as described by Liu and Ho [29]. A serial dilution of the essential oils (three concentrations of 50%, 10% and 2%) was prepared in *n*-hexane. A Whatman filter paper (diameter 2.0 cm) was impregnated with 10 µL dilution and then placed on the underside of the screw cap of a glass vial (diameter 2.5 cm, height 5.5 cm). The solvent was allowed to evaporate for 20 s before the cap was placed tightly on the glass vial, each of which contained 10 insects inside to form a sealed chamber. Fluon (ICI America Inc., London, UK) was used inside the glass vial to prevent insects from contacting the treated filter paper. *n*-Hexane was used as a control. Five replicates were carried out for all treatments and controls, and they were incubated under the same conditions for 24 h. The insects were considered dead if appendages did not move when probed with a tiny brush. The corrected mortality of essential oils was calculated according to the formula: corrected mortality (%) = 100 × (*A* − *C*)/(1 − *C*), where *A* = the mortality of treated with essential oils, *C* = the mortality of control.

### 4.5. Contact Toxicity

The contact toxicity of the essential oils against the three species of insects was tested with reference to the method of Liu and Ho [29]. The preparation of testing concentrations of the essential oils was same as the fumigant toxicity assay. Aliquots of 0.5 µL of the dilutions were applied topically to the dorsal thorax of the insects. Controls were performed using *n*-hexane. Ten insects were used for each concentration and control, and the experiment was replicated five times. Mortality was recorded after 24 h of exposure. The corrected mortality of essential oils was calculated by the same way with the fumigant toxicity assay. Range-finding surveys were run to determine the appropriate testing concentrations of the essential oil of *Z*. *dissitum* roots. A serial dilution (five concentrations) was prepared in *n*-hexane. The LD_50_ values were calculated by using Probit analysis [30]. The units were converted according to the formula: LD_50_ (μg/adult) = LD_50_ (%) × *D* (g/mL) × *V* (µL/adult) × 10^3^, where *D* = the density of the concentrated essential oil, *V* = the volume of test solution on per insect.

## 5. Conclusions

In this paper, we report the chemical composition and insecticidal activity of *Z*. *dissitum* leave and root essential oils for the first time. The results suggest that essential oil of *Z. dissitum* roots is rich in sesquiterpenoids and has potential application in the study of ecological prevention and control of storage pests. Further investigations should be focused on more detailed biological activity studies of the whole plant and/or different parts, and the safety of the essential oils to humans.

## Appendix

**Table A1:** Chemical constituents identified from the essential oils of *Z*. *dissitum* leaves and roots.

Compounds	RI *	Leaves	Roots
		Relative Content (%)	Relative Content (%)
*α*-Pinene	939	-	0.2
*β*-Pinene	979	-	0.3
Limonene	1024	-	0.5
Linalool	1094	2.1	3.8
4-Terpineol	1175	7.5	0.6
*α*-Terpineol	1181	1.5	-
Estragole	1195	-	3.9
Geraniol	1252	1.1	1.1
Piperitone	1260	-	0.4
*α*-Terpinyl acetate	1296	-	0.4
*α*-Cubebene	1350	0.9	-
Copaene	1374	2.3	-
*β*-Cubebene	1388	7.9	-
Isocaryophyllene	1410	0.4	-
Caryophyllene	1423	12.7	2.3
*Z,Z,Z*-1,5,9,9-tetramethyl-1,4,7-Cycloundecatriene	1456	3.4	8.7
*γ*-Elemene	1458	1.8	-
Alloaromadendrene	1463	0.9	-
2-Dodecanone	1491	-	0.3
*α*-Selinene	1494	-	0.2
2-Tridecanone	1496	0.8	-
*γ*-Cadinene	1513	2.1	-
*β*-Cadinene	1518	0.5	-
Calamenene	1525	1.5	-
Cadala-1(10),3,8-triene	1532	0.9	-
Diepicedrene-1-oxide	1548	-	10.7
Elemicin	1554	-	0.5
*α*-Caryophyllene	1564	-	0.1
*trans*-Nerolidol	1567	2.6	-
Germacrene D-4-ol	1574	5.7	-
Spathulenol	1577	2.7	1.0
Asarone	1580	1.1	-
Caryophyllene oxide	1584	2.4	24.0
Viridiflorol	1590	2.7	-
Humulene epoxide II	1616	-	29.4
Isoaromadendrene epoxide	1618	-	0.5
*τ*-Muurolol	1636	4.5	-
Cubenol	1643	4.3	4.1
*α*-Cadinol	1656	2.2	-
*δ*-Cadinol	1659	12.8	-
*n*-Hexadecanoic acid	1928	2.8	1.2
Monoterpenoids		12.2	7.2
Sesquiterpenoids		74.0	80.9
Others		6.0	5.9
Total identified		92.1	94.0

***** RI, retention index as determined on a HP-5MS column using the homologous series of *n*-alkanes (C_8_–C_24_).
